# Antibiotic susceptibility of cystic fibrosis lung microbiome members in a multispecies biofilm

**DOI:** 10.1016/j.bioflm.2020.100031

**Published:** 2020-06-13

**Authors:** Eva Vandeplassche, Andrea Sass, Lisa Ostyn, Mette Burmølle, Kasper Nørskov Kragh, Thomas Bjarnsholt, Tom Coenye, Aurélie Crabbé

**Affiliations:** a Laboratory of Pharmaceutical Microbiology, Ghent University, Belgium; b Department of Microbiology, University of Copenhagen, Denmark; c Costerton Biofilm Center, Department of Immunology and Microbiology, University of Copenhagen, Denmark; d Department of Clinical Microbiology, Copenhagen University Hospital, Denmark

**Keywords:** Multispecies biofilms, Antibiotics, Cystic fibrosis, Microbial diversity, Microbiota

## Abstract

The lungs of cystic fibrosis (CF) patients are often chronically colonized by multiple microbial species that can form biofilms, including the major CF pathogen *Pseudomonas aeruginosa*. Herewith, lower microbial diversity in CF airways is typically associated with worse health outcomes. In an attempt to treat CF lung infections patients are frequently exposed to antibiotics, which may affect microbial diversity. This study aimed at understanding if common antibiotics that target *P. aeruginosa* influence microbial diversity. To this end, a microaerophilic multispecies biofilm model of frequently co-isolated members of the CF lung microbiome (*Pseudomonas aeruginosa*, *Staphylococcus aureus*, *Streptococcus anginosus*, *Achromobacter xylosoxidans*, *Rothia mucilaginosa*, and *Gemella haemolysans*) was exposed to antipseudomonal antibiotics. We found that antibiotics that affected several dominant species (i.e. ceftazidime, tobramycin) resulted in higher species evenness compared to colistin, which is only active against *P. aeruginosa*. Furthermore, susceptibility of individual species in the multispecies biofilm following antibiotic treatment was compared to that of the respective single-species biofilms, showing no differences. Adding three anaerobic species (*Prevotella melaninogenica*, *Veillonella parvula*, and *Fusobacterium nucleatum*) to the multispecies biofilm did not influence antibiotic susceptibility. In conclusion, our study demonstrates antibiotic-dependent effects on microbial community diversity of multispecies biofilms comprised of CF microbiome members.

## Introduction

The lungs of cystic fibrosis (CF) patients are highly susceptible to bacterial infections [1,2]. Additionally, bacteria in the CF airways mostly persist as biofilm structures that are more tolerant to antimicrobial agents which leads to recurrent and chronic infections [3]. Subsequently, a repeated cycle of infection, hyperinflammation, and airway obstruction results in a decline of lung function [2,4]. One of the major pathogens associated with pulmonary deterioration in the CF lung is *Pseudomonas aeruginosa* [5]. Yet, this bacterium is not the sole colonizer of the CF airways and it is now clear that CF lungs contain a wide collection of microorganisms, designated as the CF lung microbiome [[6], [7], [8], [9]].

Besides the well-studied CF pathogens *P. aeruginosa*, *Staphylococcus aureus*, *Haemophilus influenzae*, and *Burkholderia cenocepacia*, the CF lung microbiome may contain other potentially pathogenic species, such as *Achromobacter xylosoxidans,* members of the *Streptococcus milleri* group, *Ralstonia* spp., *Pandorea* spp., *Stenotrophomonas maltophilia*, and *Mycobacterium* spp [7,8,10,11]. Furthermore, oral bacteria also find their way into the CF airways and become part of the bacterial community, with *Rothia mucilaginosa, Gemella haemolysans* and several anaerobic species being most frequently isolated [[11], [12], [13], [14]]. Anaerobes, such as *Prevotella* spp, *Veillonella* spp., and *Fusobacterium* spp., are increasingly being recognized as more than innocent bystanders given the growing evidence provided by culture-(in)dependent methods that they can persist in CF patients’ lungs [13,15,16]. The complex bacterial airway community evolves over a patient’s lifetime and is influenced by external factors such as antibiotic treatment [[17], [18], [19], [20]]. In older CF patients the sputum microbiome typically becomes less diverse which is associated with a decline in lung function and thus worse health outcomes [17,18].

It has been hypothesized that the bacterial species present in the CF lung microbiome can influence each other when in close proximity [[21], [22], [23], [24]]. *In vitro* experiments using polymicrobial communities, typically comprised of two to three microbiome members, have provided valuable insights in the influence of interspecies interactions on (biofilm) growth, antibiotic susceptibility, and expression of virulence factors by CF pathogens [[25], [26], [27], [28]]. However, the antibiotic susceptibility of individual bacteria in polymicrobial communities that reflect the microbial diversity of CF lungs has been poorly investigated.

In the present study we used our previously developed multispecies biofilm community model comprised of six commonly co-isolated CF microbiome members (i.e. *P. aeruginosa*, *S. aureus*, *A. xylosoxidans*, *Streptococcus anginosus*, *R. mucilaginosa*, *G. haemolysans* [29]) to study the influence of different antibiotic treatments on microbial community diversity. First, we evaluated biofilm formation of these strains in the multispecies biofilm model. Subsequently, the effect on community diversity of four antibiotics commonly used to treat *P. aeruginosa* in the CF lung was investigated. In addition, antibiotic susceptibility of individual CF microbiome members grown as multispecies biofilms was compared with that of single-species biofilms. Finally, in order to determine the potential role of anaerobes, three anaerobic species (*Prevotella melaninogenica*, *Fusobacterium nucleatum*, *Veillonella parvula*) were added to the multispecies community and antibiotic susceptibility of all species in the multispecies biofilm model was determined.

## Results & discussion

### Biofilm formation of the microaerophilic multispecies consortium

**Fig. 1:**
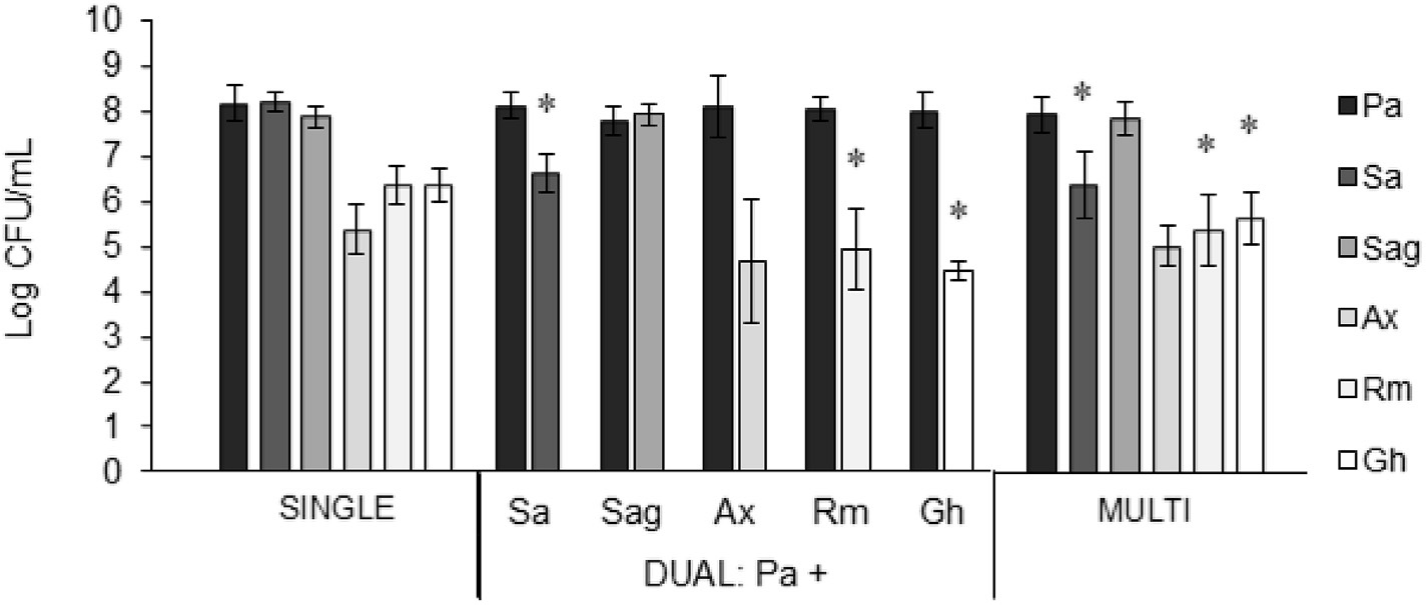
**Log CFU/mL of single-, dual-, and multispecies biofilms**. Log CFU/mL was determined by plating on selective media. Pa: *P. aeruginosa*, Sa: *S. aureus*, Sag: *S. anginosus*, Ax: *A. xylosoxidans*, Rm: *R. mucilaginosa*, Gh: *G. haemolysans*, Multi: multispecies biofilm of all six bacterial species. All graphs show means and error bars indicate standard deviations, n ​≥ ​3, ∗p ​≤ ​0.05 (compared to single-species biofilm). dual-species biofilms of *P. aeruginosa* and each individual microbiome member were cultured (Fig. 1). In dual-species biofilms *S. aureus, R. mucilaginosa* and *G. haemolysans* also grew to densities significantly (p-values: *S. aureus* 0.007, *R. mucilaginosa* 0.009, *G. haemolysans* < 0.001) lower than in the single-species biofilm which were comparable to what was observed in the multispecies biofilm. This suggests that the presence of *P. aeruginosa* was responsible for the observed inhibitory effect.

First, we evaluated whether biofilm formation was different in a single versus multispecies community. All aerobic strains formed biofilms of 5–8 log CFU/mL in the single-species biofilms as well as in the multispecies biofilm containing all six strains together (Fig. 1). In the multispecies biofilm *P. aeruginosa*, *S. anginosus*, and *A. xylosoxidans* grew to the same density as in their respective single-species biofilms. For *S. aureus*, *R. mucilaginosa*, and *G. haemolysans* the number of CFU was significantly lower in the multispecies biofilm than in the respective single-species biofilms (p-values: *P. aeruginosa* 0.196, *S. aureus* 0.001, *S. anginosus* 0.357, *A. xylosoxidans* 0.278, *R. mucilaginosa* 0.001, *G. haemolysans* 0.013). Next, we evaluated the growth of all bacterial species under the same experimental conditions as used to culture single- and multispecies biofilms (i.e. BHI ​+ ​LYS medium, microaerophilic conditions) (Fig. S1). While we observed differences in the maximal optical density reached, all species grew to stationary phase in the 48h time frame of the experiment, with the exception of *A. xylosoxidans* that reached exponential phase at the 48h time point. The slower growth of *A. xylosoxidans* could explain less biofilm formation both in single and multispecies biofilms compared to other species, such as *P. aeruginosa*.

Then, we investigated the lower density of *S. aureus*, *R. mucilaginosa*, and *G. haemolysans* in a multi-versus single-species biofilm further. The inhibitory effect of *P. aeruginosa* on *S. aureus in vitro* growth has been described [30,31] and to determine whether the lower numbers of *S. aureus, R. mucilaginosa* and *G. haemolysans* in the multispecies biofilm could be due to the presence of *P. aeruginosa*.

**Fig. 2:**
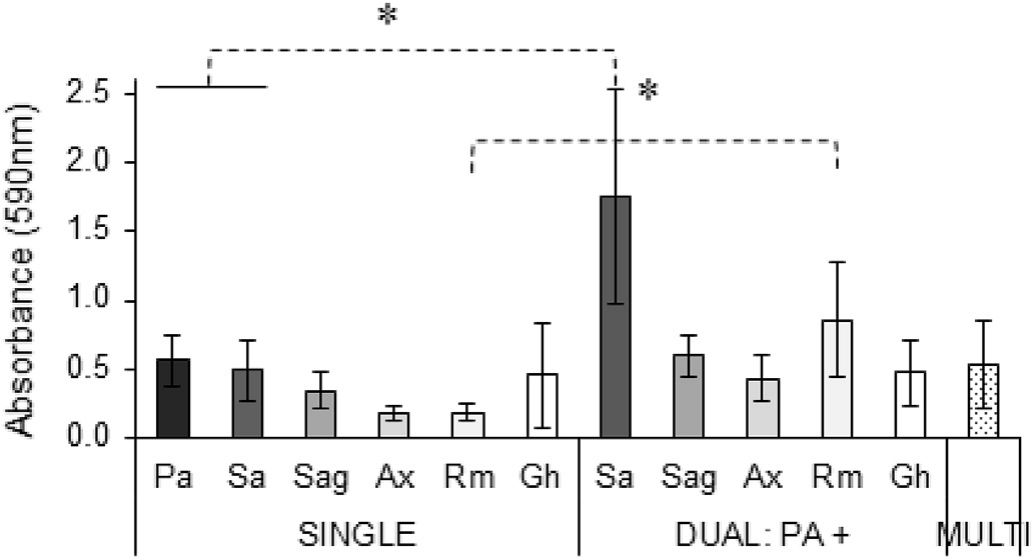
**Biomass of single-, dual-, and multispecies biofilms**. Biomass was determined by crystal violet staining. Pa: *P. aeruginosa*, Sa: *S. aureus*, Sag: *S. anginosus*, Ax: *A. xylosoxidans*, Rm: *R. mucilaginosa*, Gh: *G. haemolysans*, Multi: multispecies biofilm of all six bacterial species. All graphs show means; error bars indicate standard deviations, n ​≥ ​3, ∗p ​≤ ​0.05.

Next, we analysed the influence of growth in a multispecies community on biofilm biomass. No significant difference was observed between single- and multispecies biofilms in terms of total biomass production as assessed by crystal violet staining (Fig. 2). Hence, the previously described increased biomass production of *P. aeruginosa* and *S. aureus* mixed biofilms compared to single species biofilms was not observed in our multispecies biofilm model [23]. To determine whether differences between our study and the previous report were due to differences in experimental set-up (such as culture media, strains, oxygen levels) or community complexity we evaluated the biofilm mass of dual-species biofilms of *P. aeruginosa* and each individual species. We found a significantly higher biomass in dual-species biofilms of *P. aeruginosa* and *S. aureus* compared to that of single-cultures (Fig. 2) (p-values: *P. aeruginosa* 0.029, *S. aureus* 0.049). These data suggest that other species in the multispecies biofilm can counteract increases in biomass caused by dual-species interactions.

Taken together, these data indicate that the formation of biofilms by the six selected CF microbiome members in a multispecies biofilm occurs to a lower extent than single-species biofilms for some bacteria. The multispecies biofilm model was subsequently applied to investigate the effect of different antibiotics on community richness and evenness.

### Effect of antibiotic treatment on the diversity of a multispecies biofilm of CF lung microbiome members

**Fig. 3:**
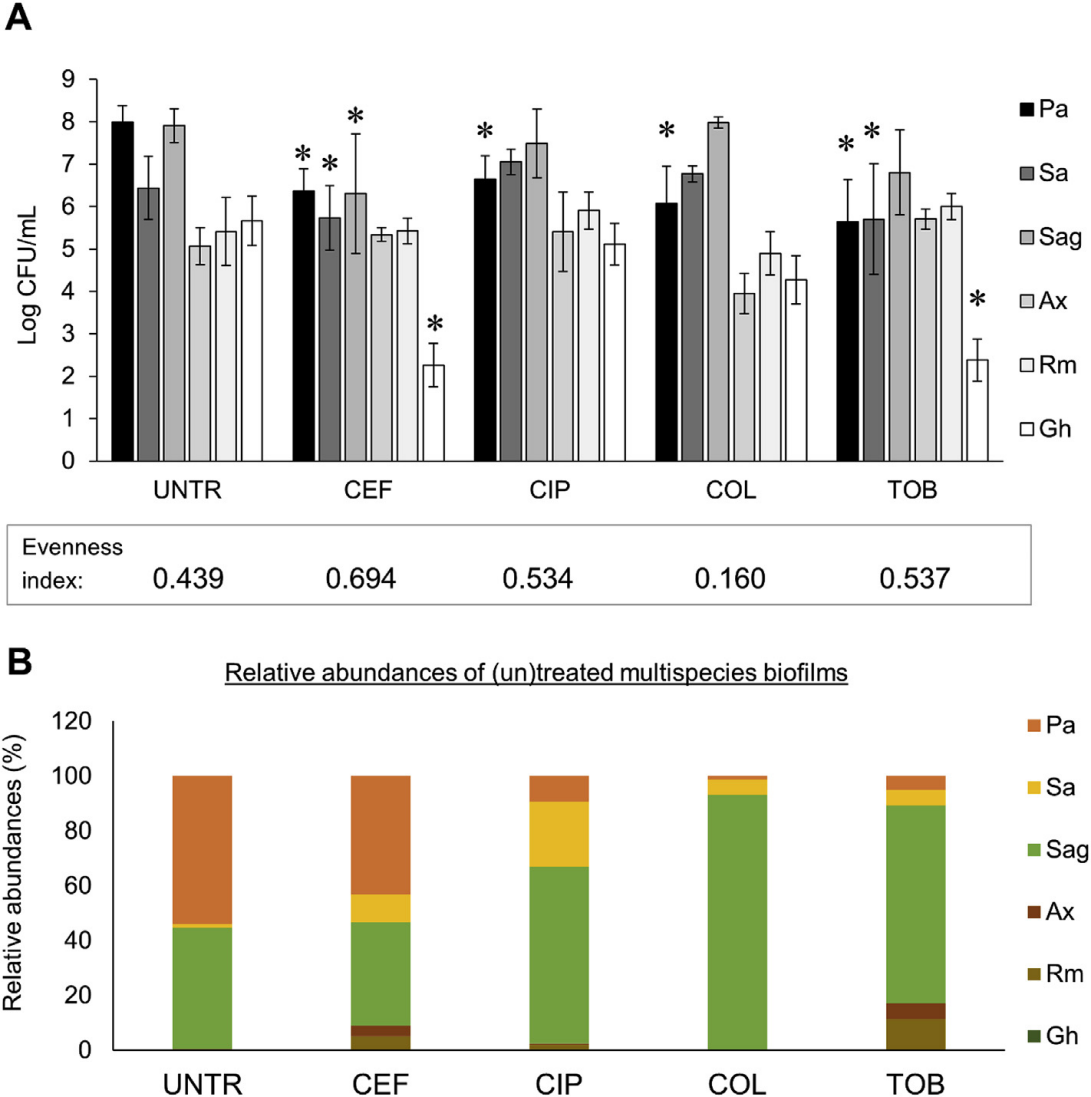
Comparison of species diversity in (un)treated multispecies biofilms: (A) Log CFU/mL values and (B) relative abundances. Species richness and evenness were assessed in four treated multispecies biofilms versus an untreated control biofilm. UNTR: untreated, CEF: 2000 ​μg/mL ceftazidime, CIP: 0.5 ​μg/mL, COL: 100 ​μg/mL colistin, TOB: 100 ​μg/mL tobramycin. Pa: *P. aeruginosa*, Sa: *S. aureus*, Sag: *S. anginosus*, Ax: *A. xylosoxidans*, Rm: *R. mucilaginosa*, Gh: *G. haemolysans.* Graphs show means and error bars indicate standard deviations, n ​≥ ​3, ∗p ​≤ ​0.05 (treated versus untreated).

The effect of different antibiotics used to treat *P. aeruginosa* infections in CF patients (i.e. ceftazidime, ciprofloxacin, colistin, tobramycin) was evaluated on the diversity of the multispecies biofilms (Fig. 3). In this study we focused on the remaining biofilm following antibiotic treatment, as the biofilm phenotype in the mucus of CF patients is believed to be the main contributor to chronic lung infections and high antibiotic tolerance [3,32,33]. We observed that all species were present before and after antibiotic treatment, hence species richness was equal for all multispecies biofilms. Species evenness is based on the relative amounts of each species present and was calculated using the Pielou’s evenness index. For the multispecies biofilms the following Pielou’s indices were calculated: untreated 0.439; ceftazidime 0.694; ciprofloxacin 0.534; colistin 0.160; tobramycin 0.537. Antibiotics that affected several dominant species resulted in a higher species evenness compared to the untreated control. This includes ceftazidime which affected the dominant pathogens *P. aeruginosa*, *S. aureus* and *S. anginosus*, and tobramycin which affected *P. aeruginosa* and *S. aureus*. In contrast, colistin, an antibiotic that was only active against *P. aeruginosa*, led to lower community evenness. The reason for the lower community evenness with colistin treatment is that another pathogen, i.e. *S. anginosus*, now dominates the community. Prior to antibiotic treatment, both *P. aeruginosa* and *S. anginosus* were the dominant species in the multispecies biofilms (Fig. 3B), a phenotype which is often observed in CF patients [34,35]. Treatment targeting *P. aeruginosa* did not affect *S. anginosus* absolute abundance (Fig. 3A) but strongly influenced the relative abundance of this microorganism (Fig. 3B). In contrast, antibiotics that eradicated both dominant pathogens *S. anginosus* and *P. aeruginosa* (ceftazidime and tobramycin) improved community diversity.

While community composition is reported to be altered (transiently) by acute antibiotic therapy in CF patients [14,[36], [37], [38], [39], [40]], the effect of specific antibiotics on microbial diversity *in vitro* or *in vivo* is mostly unexplored. Hence, *in vitro* multispecies biofilm models can serve as tools to evaluate the effect of (new) narrow/broad-spectrum antimicrobial treatments on community diversity, even though *in vivo* validation would be needed.

### Antibiotic susceptibility of CF microbial community members in a multi- and single species biofilm model

**Fig. 4:**
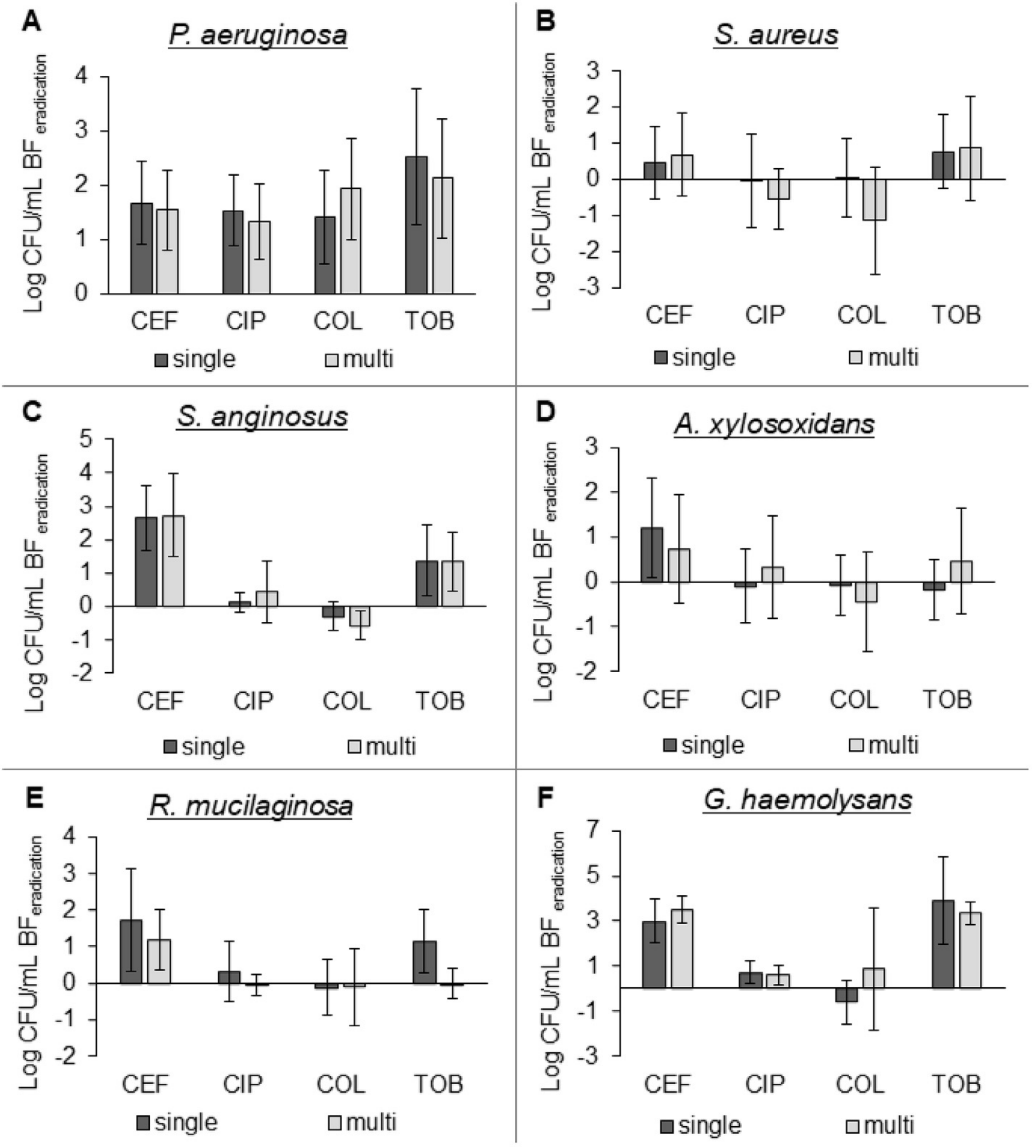
**Log CFU/mL biofilm eradication of single-versus multispecies biofilms**. Log CFU/mL determined by plating on selective media for (A) *P. aeruginosa*, (B) *S. aureus*, (C) *S. anginosus*, (D) A. *xylosoxidans*, (E) *R. mucilaginosa*, and (F) *G. haemolysans*. CEF: ceftazidime 2000 ​μg/mL, CIP: ciprofloxacin 0.5 ​μg/mL, COL: colistin 100 ​μg/mL, TOB: tobramycin 100 ​μg/mL. All graphs show means and error bars indicate standard deviations, n ​≥ ​3.

Next, we evaluated whether antibiotic susceptibility of CF microbiome members was different when grown in a single-versus multispecies biofilm. To this end, established single- or multispecies biofilms were exposed to four different antibiotics (i.e. ceftazidime, ciprofloxacin, colistin, tobramycin) and the eradication after 24h treatment was determined. For the multispecies biofilms, the data of Fig. 3 was converted to biofilm eradication. No significant differences were observed between bacterial killing in single- and multispecies biofilms (Fig. 4). However, in previous studies (with less complex communities) it has been shown that community composition does influence killing [24,[41], [42], [43], [44]]. Whether the difference between previously-obtained data and results from the present study are due to the specific taxa investigated, the antibiotics (and/or their concentration) tested, the environmental conditions and/or are related to the increase in community complexity of the multispecies biofilm compared to previous studies, remains to be determined. In addition, the isolation source of the different species and their co-evolution might also influence the experimental outcome [45]. It should be noted that in our study BHI medium supplemented with lysed blood was used for generating multispecies biofilms, which is not reflective of the CF nutritional environment. While this is a limitation of our study, accurate quantification of multispecies biofilms using plating and crystal violet assays is challenging in viscous synthetic sputum media, especially in a high through-put setting. Nevertheless, evaluation of multispecies biofilm formation and their susceptibility to antibiotics in more relevant media, such as synthetic cystic fibrosis medium 2 (SCFM2) [46] might influence the outcomes and remains to be evaluated.

### Effect of anaerobic species on antibiotic susceptibility of CF microbiome members

**Fig. 5:**
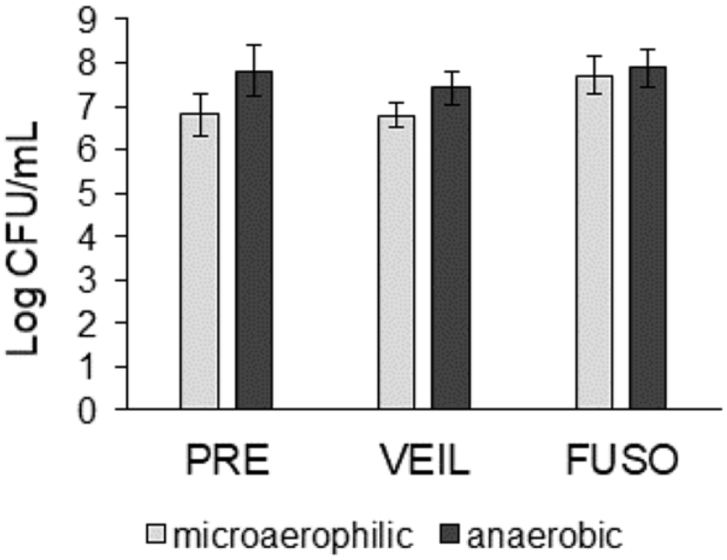
**Single-species biofilm formation (log CFU/mL) of 3 anaerobic strains in microaerophilic and anaerobic conditions.** PRE: *P. melaninogenica*. VEIL: *V. parvula*. FUSO: *F. nucleatum*. Graphs show means and error bars indicate standard deviations, n ​≥ ​3.

CF lungs are often colonized by anaerobic species, and their role in lung disease remains contradictory [13,47,48]. Therefore, we investigated if common anaerobic species of the CF lung microbiome influence the biofilm formation and antibiotic susceptibility of multispecies biofilms. To this end, the six CF strains were grown as biofilms in the presence of *P. melaninogenica*, *V. parvula*, *F. nucleatum*, or a combination of all three, and the biofilm formation and antibiotic susceptibility of each community member was determined as described before. First, to ensure that *P. melaninogenica*, *V. parvula*, and *F. nucleatum* could form biofilms in microaerophilic and anaerobic conditions, these species were grown as mono-culture biofilms (Fig. 5). We found that all three strains were able to form biofilms containing at least 7 log CFU/mL in anaerobic as well as microaerophilic conditions. This finding allowed us to add the anaerobic strains to the existing microaerophilic multispecies biofilm community model using the same experimental conditions as previously described.

Next, we assessed whether the anaerobic strains were present and grew during 48h multispecies biofilm development using qPCR analysis. An estimation of the abundance of each anaerobic strain was made based on a standard curve of DNA concentration vs. C_q_-values. At the start of the multispecies biofilm experiment, 2.5 ​× ​10^6^ ​CFU/mL of each strain was added (approx. 6.40 log CFU/mL). After 48h biofilm formation the calculated log CFU/mL values for all anaerobic strains in the multispecies biofilm were similar to (for *P. melaninogenica* and *F. nucleatum*) or higher than (for *V. parvula*) this inoculum (Table 1). These data indicate that all anaerobic strains were present, and that for *V. parvula* growth occurred, over the 48h incubation period. Nevertheless, we cannot exclude that a part of the detected DNA was derived from non-viable bacteria or extracellular DNA, leading to a possible overestimation of the estimated log CFU/mL in the multispecies biofilm.

**Table 1:** C_q_-values for *P. melaninogenica*, *V. parvula*, and *F. nucleatum* (and negative controls) in the multispecies biofilm (obtained via qPCR) and the estimated log CFU/mL (based on C_q_-values).

Species name	Mean C_q_-value	StDev	Negative control	Estimated log ​CFU/mL
*P. ​melaninogenica*	20.09	1.13	37.59	6.51
*V. ​parvula*	14.42	2.26	No signal	7.16
*F. ​nucleatum*	14.62	0.85	No signal	6.64

StDev: standard deviation. No signal ​= ​C_q_-value > 40.00.

**Fig. 6:**
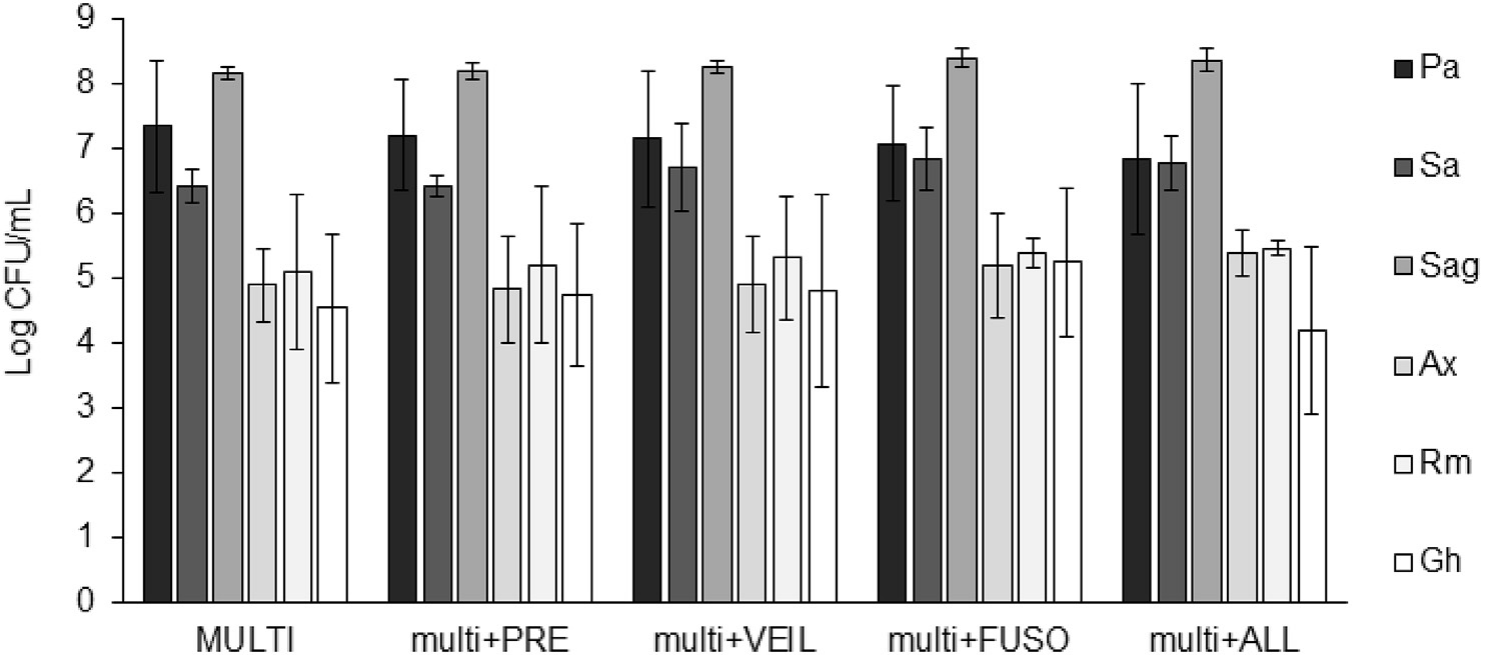
**Biofilm formation (log CFU/mL) of multispecies biofilms in the presence of anaerobic species. MULTI: multispecies biofilm of six aerobic and microaerophilic strains**. Pa: *P. aeruginosa*, Sa: *S. aureus*, Sag: *S. anginosus*, Ax: *A. xylosoxidans*, Rm: *R. mucilaginosa*, Gh: *G. haemolysans.* PRE: *P. melaninogenica*, VEIL: *V. parvula*, FUSO: *F. nucleatum*. ALL: *P. melaninogenica*, *V. parvula*, and *F. nucleatum*. Graphs show means and error bars indicate standard deviations, n ​≥ ​3.

**Fig. 7:**
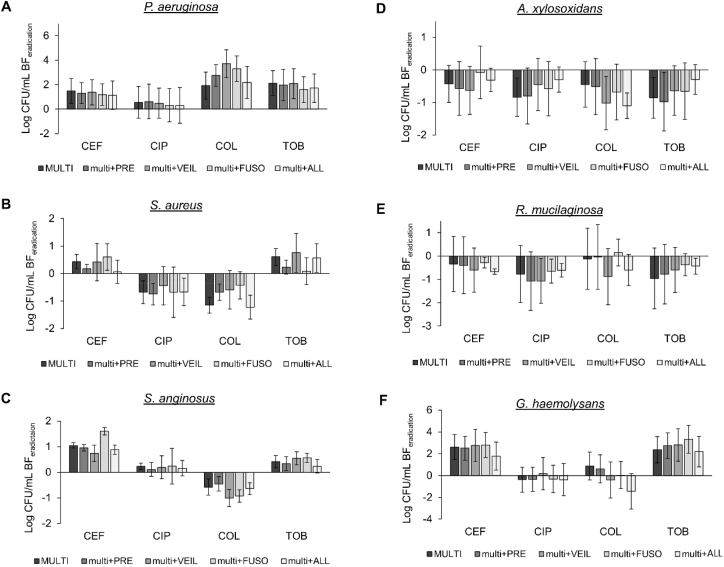
Log CFU/mL biofilm eradication of (A) *P. aeruginosa*, (B) *S. aureus*, (C) *S. anginosus*, (D) *A. xylosoxidans*, (E) *R. mucilaginosa*, and (F) *G. haemolysans* in the presence of anaerobic strains, after treatment with ceftazidime (CEF), ciprofloxacin (CIP), colistin (COL), or tobramycin (TOB). MULTI: multispecies biofilm of six aerobic and microaerophilic strains. PRE: *P. melaninogenica*. VEIL: *V. parvula*. FUSO: *F. nucleatum*. ALL: *P. melaninogenica*, *V. parvula*, and *F. nucleatum*. All graphs show means and error bars indicate standard deviations, n ​≥ ​3.

Next, biofilm formation and antibiotic susceptibility of all six strains was evaluated in the multispecies biofilms in the presence of *P. melaninogenica*, *V. parvula*, or *F. nucleatum*, or a combination of all three. None of the aerobic or microaerophilic biofilm community members were influenced by the presence of anaerobic strains (Fig. 6, Fig. 7).

Anaerobic species have increasingly become the subject of research with the discovery that they are abundant in the CF lung microbiome [13,15,16] and may influence other members of the microbial community [13]. For example, Sherrard *et al* [49]. showed that *Prevotella* spp. can secrete extended-spectrum β-lactamases, hereby protecting *P. aeruginosa* from the antimicrobial action of ceftazidime. Additionally, Luppens *et al* [50]. found that in a dual-species biofilm of *V. parvula* with *Streptococcus mutans* the latter showed increased survival after various antimicrobial treatments (amine chloride, zinc chloride, erythromycin, or hydrogen peroxide). In the context of dental plaque co-cultivation of *Veillonella* spp. and *Streptococcus* spp., and *S. mutans* and *F. nucleatum* led to specific co-aggregation, resulting in facilitated early biofilm formation [51,52].

Enhancing the complexity of *in vitro* multispecies communities, hereby increasing physiological relevance, may thus provide a different experimental outcome in comparison to co-cultures of two or three bacterial species [28]. Although community complexity in the present study is enhanced compared to previous reports, the multispecies biofilm model is still composed of a limited number of species and contains multiple pathogens. Hence, the effect of antibiotic treatment on the diversity of a richer and more diverse bacterial community may have a different experimental outcome. Furthermore, as mentioned earlier differences in experimental set-up (culture medium, strains, antibiotic concentrations, oxygen levels) should also be considered. Finally, our experimental approach that studied the response of the community as a whole does not capture spatial heterogeneity of the biofilm [53], where local interspecies interactions could potentially lead to effects on antibiotic susceptibility.

In conclusion, exposure of multispecies biofilms to different antibiotics that are commonly used to treat *P. aeruginosa* lung infections in CF patients revealed antibiotic-dependent effects on microbial community evenness. We observed that antibiotics that simultaneously targeted other dominant pathogens besides *P. aeruginosa* led to a community with higher evenness as compared to antibiotics that were only active against *P. aeruginosa*. In addition, the antibiotic susceptibility of individual members in the CF biofilm community was comparable to that of single species biofilms, indicating that the complex community did not influence antibiotic efficacy under our experimental conditions.

This study highlights that controlled *in vitro* multispecies models can provide information on the influence of external disturbances, such as antibiotic treatment, on microbiome composition and diversity. Insights from these types of model platforms may be useful to fine-tune antimicrobial treatment strategies in ways that restore and/or limit effects on microbial diversity while targeting key CF pathogens.

## Materials and methods

### Bacterial strains and culturing conditions

Strains used in this study are listed in Table 2, and were first cultured from −80 ​°C glycerol stocks onto suitable solid media. Liquid cultures were then grown until stationary phase at 37 ​°C while shaking at 250 ​rpm in BHI broth, with supplementation of 2.5% lysed blood (Biotrading; protocol for lysed horse blood according to EUCAST) [54] (BHI ​+ ​LYS) for the anaerobic strains (Table 2). *S. anginosus* and *G. haemolysans* cultures were incubated in microaerophilic conditions (±5% O_2,_ ±15% CO_2_; CampyGen Compact system, Thermo Fisher Scientific, USA) while *P. melaninogenica*, *V. parvula*, and *F. nucleatum* cultures were incubated in anaerobic conditions (AnaeroGen Compact system, Thermo Fisher Scientific).

**Table 2:** Overview of strains and culturing conditions used in this study.

Species name	Strain number	Source	Liquid culture medium	Solid medium
*Pseudomonas ​aeruginosa*	AA2 ​= ​LMG27630	CF lung (early infection)	BHI	LB
*Staphylococcus aureus*	SP123	Sputum (Belgium) [55]	BHI	LB
*Streptococcus ​anginosus*	LMG14696	Respiratory tract	BHI	BHI
*Achromobacter ​xylosoxidans*	LMG26680	Sputum CF patient (Belgium)	BHI	NA
*Rothia mucilaginosa*	DSM20746	Throat	BHI	NA
*Gemella haemolysans*	LMG18984	Sputum	BHI	CBA
*Prevotella ​melaninogenica*	DSM7089	Sputum	BHI ​+ ​LYS	CBA
*Veillonella parvula*	DSM2007	Mouth	BHI ​+ ​LYS	CBA
*Fusobacterium ​nucleatum*	DSM19507	Periodontal pocket	BHI ​+ ​LYS	CBA

BHI: Brain Heart Infusion broth/agar; LYS: lysed horse blood; LB: Luria Bertani agar; NA: Nutrient agar; CBA: Columbia Blood Agar (Columbia agar base ​+ ​5% sheep blood).

### Growth curves

Bacterial liquid cultures were grown to stationary phase as described above, and diluted to 5 ​× ​10^7^ ​CFU/mL (based on OD_590nm_) in BHI ​+ ​LYS medium. Diluted cultures were transferred to 96-well plates (100 μL/well) and incubated statically for 48 ​h ​at 37 ​°C in an EnVision Multilabel Plate Reader (PerkinElmer, USA), and OD_590nm_ was measured every 30 ​min. To generate microaerophilic conditions, the outer wells of the plate were filled with ascorbic acid from CampyGen Compact System (Thermofisher Scientific, USA), and the plate was sealed with silicone.

### Formation of single- and multispecies biofilms

Biofilms were grown in a PVC flat-bottomed 96-well microtiter plate (Thermo Fisher) as described previously in BHI ​+ ​LYS medium [29]. While the composition of this medium does not reflect the CF nutritional environment, it was used since all selected species survived and/or grew in this medium for the duration of the experiment, and allowed selective quantification of the biofilm fraction and biomass using conventional protocols. Briefly, stationary phase liquid cultures were diluted in BHI ​+ ​LYS to approx. 5 ​× ​10^7^ ​CFU/mL (based on OD_590nm_). In all conditions, an equal number of cells per strain (2.5 ​× ​10^6^ ​CFU, i.e. 50 ​μL) was inoculated while the final volume in each well was 100 ​μL, which was obtained by concentrating the samples via centrifugation. All 96-well plates were incubated in a microaerophilic environment (±5% O_2,_ ±15% CO_2_; CampyGen Compact system), thereby mimicking the low oxygen conditions in the CF lung [56,57], at 37 ​°C for 24 ​h.

### Effect of antibiotic exposure on single- and multispecies biofilms

24 ​h old biofilms were exposed to antibiotics for an additional 24 ​h as described previously [29], where after the abundance of each species was determined by plating on selective media [29]. Briefly, the biofilms were first rinsed with physiological saline solution (PS; 0.9% [w/v] NaCl in milliQ water). Subsequently, 100 ​μL of medium (BHI ​+ ​LYS) or 100 ​μL of antibiotic solution in the same medium was added to the untreated (control) and treated biofilms, respectively. Four antibiotics from four major antibiotic classes frequently used to treat *P. aeruginosa* in CF were selected: ceftazidime (2000 ​μg/mL, 125x MIC *P. aeruginosa*), ciprofloxacin (0.5 ​μg/mL, 2x MIC *P. aeruginosa*), colistin (100 ​μg/mL, 25x MIC *P. aeruginosa*), and tobramycin (100 ​μg/mL, 100x MIC *P. aeruginosa*). Antibiotic concentrations were chosen to obtain *P. aeruginosa* killing of around 2–3 log CFU/mL, based on optimisation experiments. MICs of these antibiotics for *P. aeruginosa*, *S. aureus*, *S. anginosus*, *A. xylosoxidans*, *R. mucilaginosa*, and *G. haemolysans* were determined using EUCAST guidelines [54], and are presented in Table S1. All biofilms were incubated under microaerophilic conditions at 37 ​°C for 24 ​h. Subsequently, biofilms were rinsed with physiological saline to remove planktonic cells before quantification. To quantify the number of colony forming units (CFU) of *P. aeruginosa*, *S. aureus*, *S. anginosus*, *A. xylosoxidans*, *R. mucilaginosa*, and *G. haemolysans*, biofilms were homogenized by two rounds of vortexing (900 ​rpm, 5 ​min) and sonication (5 ​min; Branson Ultrasonic bath, Hach Company, USA), and plated on six selective media, as described previously [29]. To investigate antibiotic susceptibility of biofilms, the log CFU/mL value for the treated single- or multispecies biofilm was subtracted from the log CFU/mL value from the untreated single- or multispecies biofilms. This resulted in a ‘log CFU/mL eradication’ value. Finally, biomass of biofilms was quantified by crystal violet staining as described elsewhere [58].

### Pielou’s evenness index calculation

Diversity indices represent a combination of richness and evenness components. The richness of a community is based on the number of different species that are present, while the evenness shows how similar the relative quantities of the species are [59]. Pielou’s evenness index (J′) is based on the Shannon diversity index and is given by the following formula:

(1) $$\text{J}’\;=\;\frac{H’}{H’max}$$

where H′ is the Shannon diversity index [H ​= ​∑ - (Pi ∗ ln Pi) (with Pi ​= ​proportional abundance)], H’_max_ ​= ​ln(S) and S is the total number of species. J′ has a value between 0 and 1; 0 being the most uneven and 1 the most even community [59,60].

### DNA extraction from planktonic and biofilm cultures

For single-species planktonic cultures, 2 ​mL of stationary phase cultures were centrifuged and the pellet resuspended in TE buffer (200 ​μL; 10 ​mM Tris-HCl pH 8; 1 ​mM EDTA pH 8). The resuspended pellet was pre-treated with 20 ​mg/mL lysozyme (Sigma-Aldrich, USA) and 0.1 ​mg/mL proteinase K (Sigma-Aldrich) at 37 ​°C for 1 ​h. Then, 100 ​μL of this suspension was added to ‘lysis tubes’ containing 500 ​μL acid-washed glass beads (Sigma-Aldrich) and 500 ​μL lysis buffer (50 ​mM Tris-HCl pH 8, 70 ​mM EDTA pH 8, 1% sodium dodecyl sulphate) supplemented with 0.5 ​μg/mL pronase (Roche, Germany). After vortexing, the tubes were incubated at 37 ​°C for 1 ​h and then centrifuged (13,000 ​rpm). Saturated ammonium acetate (200 ​μL) was added, the mixture was vortexed again, and centrifuged for 2 ​min. Then, chloroform (600 ​μL) was added, vortexed, and centrifuged for 5 ​min to separate the phases. Afterwards, 400 ​μL of the top phase was transferred to a tube containing 1 ​mL of 100% ethanol. After mixing by inversion, this was centrifuged for 5 ​min. The supernatant was removed, the pellet washed with 70% ethanol, and then air-dried. Resulting DNA was dissolved in low-EDTA-TE buffer [10 ​mM Tris-HCl pH 8; 0.1 ​mM EDTA pH 8; 0.5 ​μg/mL RNase A (Qiagen, Germany)]. DNA of multispecies biofilms was extracted using the PureLink Microbiome DNA extraction kit (Thermo Fisher Scientific) according to the manufacturer’s instructions. DNA concentrations were measured using the BioDrop μLITE (Isogen Life Science, The Netherlands).

### Quantitative real-time PCR

To confirm the presence of *P. melaninogenica*, *V. parvula*, and *F. nucleatum* in the multispecies biofilms, quantitative real-time PCR (qPCR) (C1000 Touch Thermal Cycler, Bio-rad) was performed using primers targeting the 16S rRNA gene (Table 3). Primers for *V. parvula* and *F. nucleatum* were previously described [61]. For *P. melaninogenica*, primers targeting the V1 and V2 hypervariable regions of the 16S rRNA genes were designed using Primer3Web version 4.1.0 (http://primer3.ut.ee/) using the following parameters: optimal primer size 20 (range: 18 to 27), optimal T_m_ 60 (range 57–63) and GC content from 20 to 80%. Primers were then blasted against the genomes of all other eight species, to rule out sequence complementarity. A common annealing temperature (58 ​°C) for all primers was determined via temperature gradient qPCR.

**Table 3:** Real-time PCR primers used for *P. melaninogenica*, *V. parvula*, and *F. nucleatum*.

Species name	Oligonucleotide sequence	Amplicon size (bp)	E
*Prevotella melaninogenica*	F: 5’ - CTTGCACTCTTTGGACGTCGAC - 3′ R: 5’ - TTACTTCAAATCTGATGCCGTCATCG - 3′	133	1.76
*Veillonella parvula*	F: 5’ - ATCAACCTGCCCTTCAGA - 3′ R: 5’ - AAGCTCTGTTAATCGGGACG - 3′	343	1.84
*Fusobacterium nucleatum*	F: 5’ - CAAACGCGATAAGTAATC - 3′ R: 5’ - CCCTTTCGTATGTTACCA - 3′	273	1.94

E ​= ​amplification efficiency. An efficiency of 2.00 is equivalent to 100% of theoretical amplification per PCR cycle.

Each qPCR reaction (total volume of 20 ​μL) contained 1 ​μL salmon sperm DNA (25 ​ng/μL; Sigma-Aldrich), 0.6 ​μL primer mix (Sigma-Aldrich; 10 ​μM), 10 ​μL Perfecta SYBR Green Fast mix (Quanta bio, USA), and 2 ​μL template DNA (25 ​ng/μL). Non-target salmon sperm DNA was added to all samples to prevent sample loss from binding to (low-bind) tubes.

Specificity of the primers was confirmed by PCR, followed by gel electrophoresis, and by melting curve analysis. Amplification efficiency (E) was determined using the following formula: E ​= ​10^(-1/slope) (with slope being the slope of the dilution curve obtained with 10-fold dilutions), and correlations with equation y ​= ​−4.08x ​+ ​18.21 (R^2^ ​= ​0.9994), y ​= ​−3.78x ​+ ​15.16 (R^2^ ​= ​0.9979), y ​= ​−3.47x ​+ ​15.51 (R^2^ ​= ​0.9786) were obtained for *P. melaninogenica*, *V. parvula* and *F. nucleatum*, respectively. qPCR reaction efficiency in the presence or absence of non-target bacterial DNA was compared by composing a standard curve of various target DNA concentrations (1/5 dilutions; 500–0.8 ​pg/μL) with or without a DNA mix of all other species (*P. aeruginosa*, *S. aureus*, *S. anginosus*, *A. xylosoxidans*, *R. mucilaginosa*, *G. haemolysans*, and the two non-target anaerobes, with a total final concentration of 1 ​ng/μL in the reaction), resulting in a standard curve of DNA concentration vs. C_q_-values (Supplementary Fig. S2). The slope and individual C_q_-values for each sample were not affected by the presence of microbiome members. Finally, the C_q_-value for each anaerobic strain in the multispecies biofilm was determined (Table 3) and used to estimate the log CFU/mL of each of the anaerobic strains in the multispecies biofilm (Table 3 & Supplementary Fig. S3). This approach allowed to estimate the abundance of the anaerobic strains in the multispecies biofilms.

### Statistical analysis

All experiments were performed in at least biological triplicate. Statistical analysis was performed in SPSS 24.0. First, normality of the data was examined via the Shapiro-Wilk test. Normally distributed data were then assessed by a two-tailed independent samples *t*-test or one-way ANOVA followed by a Dunnett’s post hoc analysis. Not normally distributed data were evaluated using a Mann-Whitney or Kruskal-Wallis non-parametric test. Statistical significance of data is concluded when p-values are ≤0.05.

## Data availability

All data generated or analysed during this study are included in this published article (and its Supplementary Information files).

## CRediT authorship contribution statement

A.C. and E.V. conceptualized the study. E.V., A.S. and A.C. designed the experimental set-up. E.V. and L.O. performed all experiments. E.V., A.C., T.C., T.B., K.N.K. and M.B. were involved in data analysis. E.V., A.C. and T.C. wrote the main manuscript text with input from all authors. All authors reviewed the manuscript.

## Declaration of competing interest

The authors declare no competing interests.
