# Supplementary material for: Antibiotic susceptibility of cystic fibrosis lung microbiome members in a multispecies biofilm
Source: Biofilm. 2020 Jun 13;2:100031. doi: 10.1016/j.bioflm.2020.100031 (PMC7798459; doi:10.1016/j.bioflm.2020.100031)
Supplement: Multimedia component 1 [file mmc1.pdf]

SUPPLEMENTARY INFORMATION

Antibiotic susceptibility of cystic fibrosis lung microbiome members in a multispecies biofilm

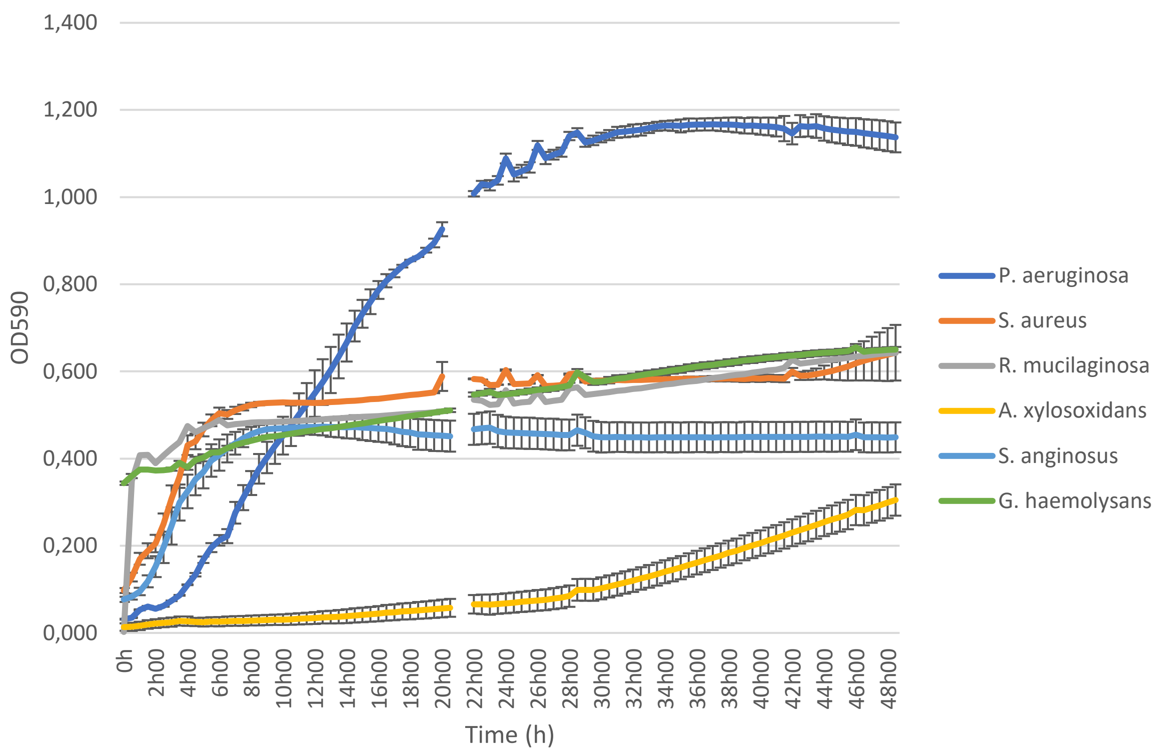

Supplementary Figure S1. Growth curves of *P. aeruginosa*, *S. aureus*, *R. mucilaginosa*, *A. xylosoxidans*, *S. anginosus* and *G. haemolysans* in BHI + LYS medium under microaerophilic (5% O_2) conditions. Graph shows means; error bars indicate standard error; n = 3.

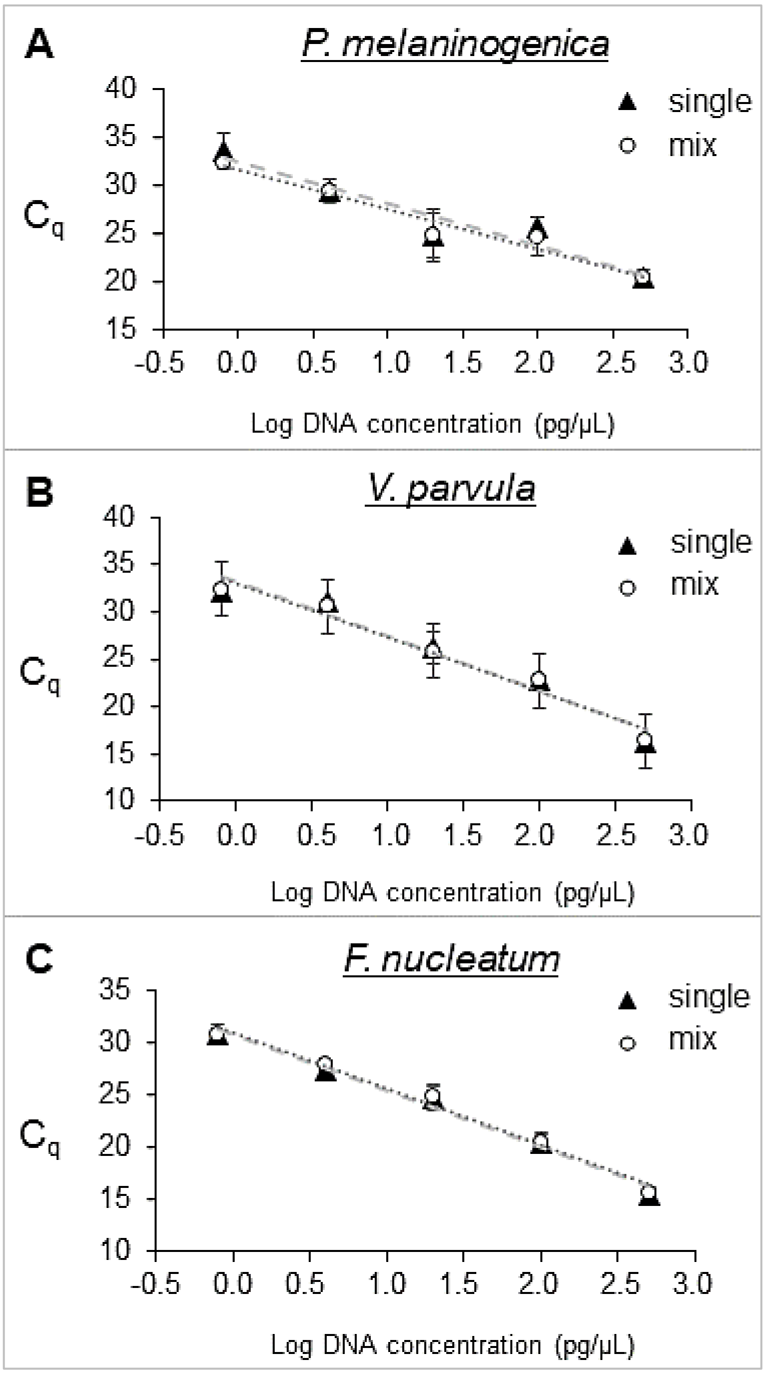

Supplementary Figure S2. Standard curves to validate qPCR reaction efficiency for (A) *P. melaninogenica*, (B) *V. parvula*, and (C) *F. nucleatum* primers at various (lower) DNA concentrations, in the presence or absence of non-target DNA. Single: only target DNA was present at various concentrations. Mix: non-target DNA of all other bacterial strains (*P. aeruginosa*, *S. aureus*, *S. anginosus*, *A. xylosoxidans*, *R. mucilaginosa*, *G. haemolysans*, and the two non-target anaerobic strains) was added to the same concentrations of target DNA, to confirm that no differences in qPCR reaction efficiency occurred in the presence of non-target DNA. Graphs show means and error bars indicate mean absolute deviations, n = 2.

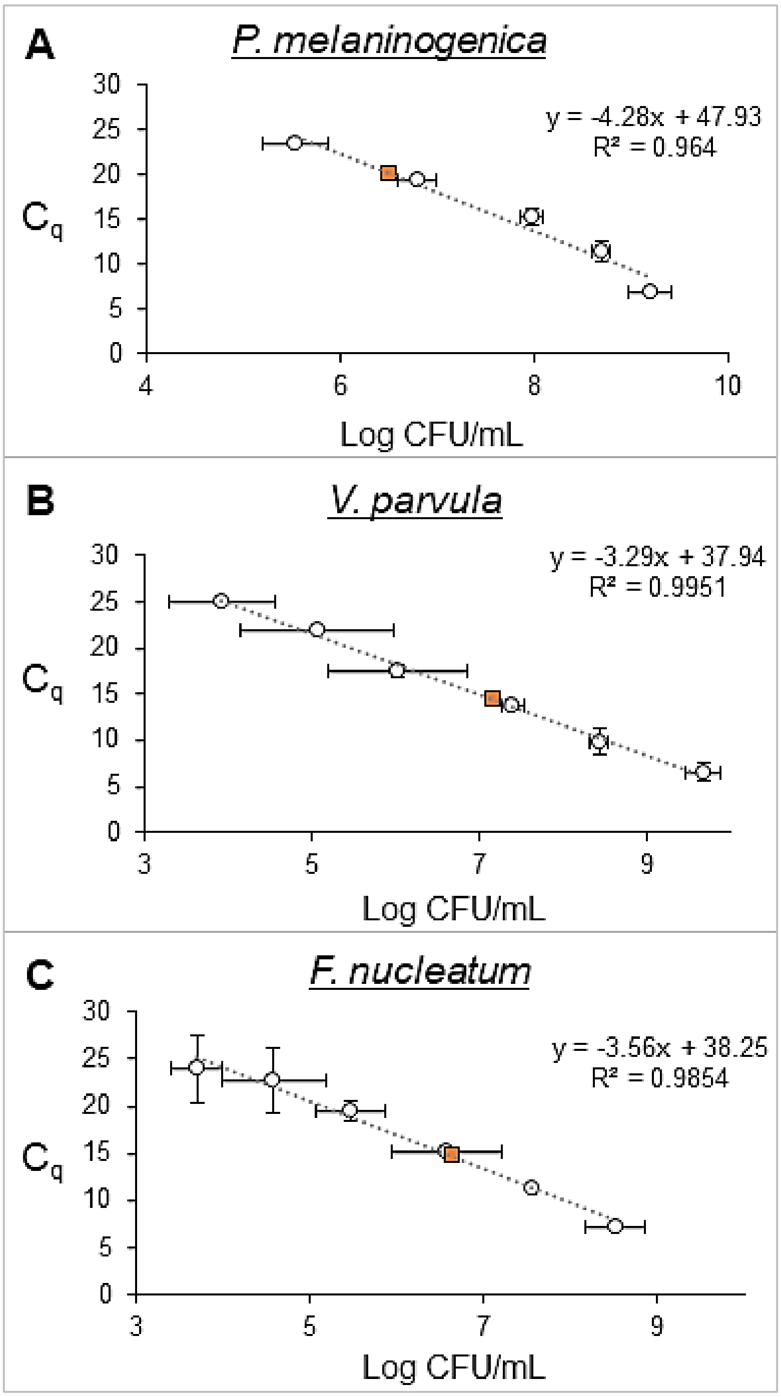

Supplementary Figure S3. Log CFU/mL versus C_q-value standard curves for (A) *P. melaninogenica*, (B) *V. parvula*, and (C) *F. nucleatum*. Log CFU/mL was determined via plating and C_q-values via qPCR. Orange squares (□) indicate C_q-values found in the multispecies biofilm samples. Graphs show means and error bars indicate mean absolute deviations, n = 2.
