# Supplementary material for: Antibiotic susceptibility of cystic fibrosis lung microbiome members in a multispecies biofilm
Source: Biofilm. 2020 Jun 13;2:100031. doi: 10.1016/j.bioflm.2020.100031 (PMC7798459; doi:10.1016/j.bioflm.2020.100031)
Supplement: Multimedia component 2 [file mmc2.docx]

| Species | MIC | | | | |
| --- | --- | --- | --- | --- | --- |
|  | | CEF | CIP | COL | TOB |
| *Pseudomonas aeruginosa* | | 16 | 0.25 | 4 | 1 |
| *Staphylococcus aureus* | | >256 | 64 | 128 | 2 |
| *Streptococcus anginosus* | | 2 | 1 | >256 | 64 |
| *Achromobacter xylosoxidans* | | 4 | 4 | >256 | >256 |
| *Rothia mucilaginosa* | | 8 | 16 | 128 | 32 |
| *Gemella haemolysans* | | 1 | 0.5 | >256 | 2 |

Table S1. MIC of CF microbiome members used to include in multispecies biofilms

CEF: ceftazidime, CIP: ciprofloxacin, COL: colistin, TOB: tobramycin.
